# Functional dependence as a contributing factor for patient hand contamination by multidrug-resistant organisms in acute care

**DOI:** 10.1017/ash.2023.320

**Published:** 2023-09-29

**Authors:** Trenton Behunin, Julia Mantey, Marco Cassone, Lona Mody

## Abstract

**Background and objectives:** Patients with functional disabilities are at higher risk of adverse outcomes, including infections. Although healthcare worker hand contamination has long been recognized as an important source of pathogen acquisition, the role of patient hands has been less clearly defined. We sought to determine whether the presence of functional disabilities is correlated with patient hand contamination by multidrug-resistant organisms (MDROs) and thus a potential target for patient hand hygiene (PHH) interventions. **Methods:** Case–control study of hand contamination with methicillin-resistant *S. aureus*, vancomycin-resistant enterococci, and gram-negative bacilli resistant to cephalosporins, fluoroquinolones, and/or carbapenems in 2 acute-care hospitals in southeastern Michigan. Cases (n = 40) and controls (n = 359) were defined as patients with or without hand contamination by MDROs, respectively. We assigned 3 exposure categories based on Katz activities of daily living scores: no functional disabilities (independent, reference group), 1–3 functional disabilities (partially dependent), and 4+ functional disabilities (dependent). We used stepwise logistic regression to identify confounding variables. Logistic regression was then used to establish the relationship between a patient’s functional dependence level and their hand contamination by MDROs. **Results:** The distribution of hand contamination of each target MDRO by level of patient dependence is shown in the Table. Overall, methicillin-resistant *Staphylococcus aureus* (MRSA) was the most represented, followed by resistant gram-negatives and vancomycin-resistant enterococci (VRE). Hospital site, sex, and history of MDROs were included in the model based on stepwise regression. The odds ratio (OR) of MRSA hand contamination in the dependent category was 3.19 (95% CI, 1.18–5.54) compared to the independent category, and for any MDRO the OR was 2.77 (95% CI, 1.22–6.32) (Fig. 1). The OR of MRSA hand contamination in the partially dependent category was 1.38 (95% CI, 0.27–7.07) compared to the independent group, and for any MDRO the OR was 0.86 (95% CI, 0.24–3.10) (Fig. 1). Feeding dependence had the highest single association with hand contamination (OR, 3.79, 95% CI, 1.26–11.43), with dressing dependence having the second highest association with hand contamination (OR, 2.82; 95% CI, 1.31–6.05) (Fig. 2). **Conclusions:** Patients with more functional dependencies were more likely to have MDRO hand contamination. This finding suggests a need for targeted PHH interventions in patients with functional disabilities to help prevent the spread of MDROs in the acute-care setting.

**Figure:**
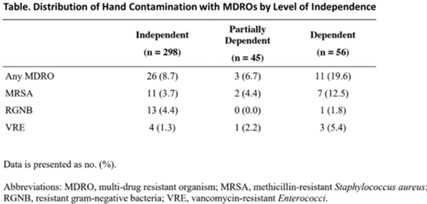

**Figure:**
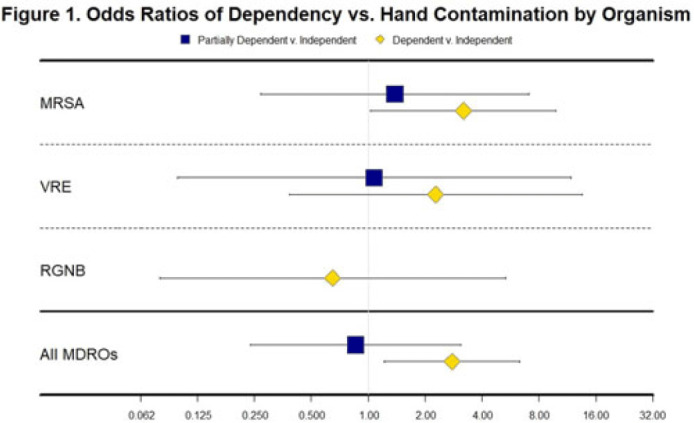

**Figure:**
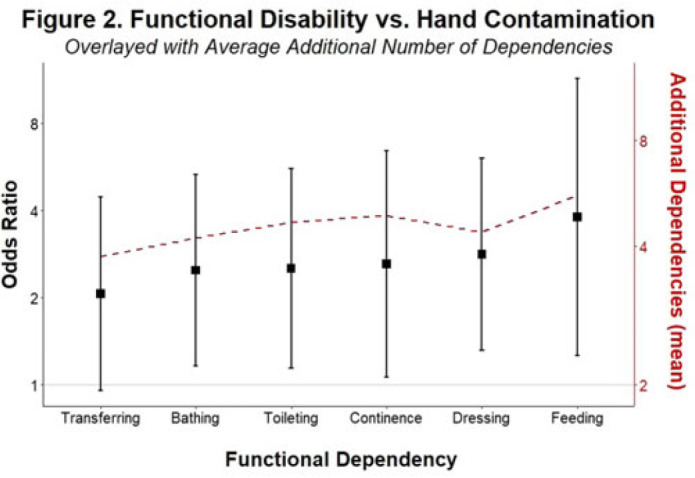

**Disclosures:** None

